# Interaction of Soy Protein Isolate Hydrolysates with Cyanidin-3-*O*-Glucoside and Its Effect on the In Vitro Antioxidant Capacity of the Complexes under Neutral Condition

**DOI:** 10.3390/molecules26061721

**Published:** 2021-03-19

**Authors:** Yaru Wu, Zhucheng Yin, Xuejiao Qie, Yao Chen, Maomao Zeng, Zhaojun Wang, Fang Qin, Jie Chen, Zhiyong He

**Affiliations:** 1 State Key Laboratory of Food Science and Technology, Jiangnan University, Wuxi 214122, Jiangsu, China; yaru_wu828@163.com (Y.W.); 15921730158@163.com (Z.Y.); qiexjhb@163.com (X.Q.); chenyaomail666@163.com (Y.C.); mmzeng@jiangnan.edu.cn (M.Z.); zhaojun.wang@jiangnan.edu.cn (Z.W.); qfflast@sina.com (F.Q.); Chenjie@jiangnan.edu.cn (J.C.); 2 International Joint Laboratory on Food Safety, Jiangnan University, Wuxi 214122, Jiangsu, China

**Keywords:** soy protein isolate, hydrolysate, cyanidin-3-*O*-glucoside, noncovalent interaction, antioxidant capacity

## Abstract

The interaction of soy protein isolate (SPI) and its hydrolysates (SPIHs) with cyanidin-3-*O*-glucoside (C3G) at pH 7.0 were investigated to clarify the changes in the antioxidant capacity of their complexes. The results of intrinsic fluorescence revealed that C3G binds to SPI/SPIHs mainly through hydrophobic interaction, and the binding affinity of SPI was stronger than that of SPIHs. Circular dichroism and Fourier-transform infrared spectroscopy analyses revealed that the interaction with C3G did not significantly change the secondary structures of SPI/SPIHs, while the surface hydrophobicity and average particle size of proteins decreased. Furthermore, the SPI/SPIHs-C3G interaction induced an antagonistic effect on the antioxidant capacity (ABTS and DPPH) of the complex system, with the masking effect on the ABTS scavenging capacity of the SPIHs-C3G complexes being lower than that of the SPI-C3G complexes. This study contributes to the design and development of functional beverages that are rich in hydrolysates and anthocyanins.

## 1. Introduction

In recent years, consumers have flocked to beverages that combine soymilk and fruit juice due to their good flavor, nutritional properties, and health benefits [1,2]. Fruits contain a variety of polyphenols, a type of phytoactive compound thought to reduce the risk of some diseases, such as obesity, cardiovascular and neurodegenerative diseases [3]. Soy provides the main protein in these fruit juice–soymilk beverages, which is of high-quality and contains all the essential amino acids, and less saturated fat and no cholesterol compared with animal sources of protein [4]. Therefore, the polyphenols in fruit and soy protein inevitably interact in fruit juice–soymilk beverages. Recently, several studies have demonstrated that the protein–polyphenol interactions changed the structure, antioxidant activity, stability, and in vitro digestibility of soy protein [5,6]. A number of studies have indicated that the addition of polyphenols can increase the antioxidant capacity of polyphenols–soy protein isolate (SPI) conjugates [6,7]. However, some results suggest that the radical scavenging activity values of protein–polyphenol complexes are less than the sum of their individual values, indicating that partial free antioxidant groups are masked because of the protein–polyphenol interactions [5,8].

Compared with soy protein, its hydrolysates (SPIHs) exhibit various beneficial properties such as good solubility and metal chelating capacity [9]. Due to the presence of soy bioactive peptides, which are small and specific fragments of the soy protein mainly derived from enzymatic hydrolysis [4,10], the antioxidant capacity of the hydrolysate is improved. In novel beverages containing protein hydrolysates and polyphenols, it is possible that peptides and polyphenols interact, but only a few researchers have focused on these types of interactions [11]. Previous studies have found that curcumin delivery vehicles constructed with soy protein hydrolysates or γ-zein hydrolysates enhanced sustained release, encapsulation efficiency, and physicochemical stability of curcumin [12,13]. In one study, Milea et al. [14] improved the functional properties of flavonoids extracted from yellow onion skins through freeze-drying microencapsulation that was obtained with whey protein hydrolysates and different polymer-based coating materials. Moreover, some studies revealed that the number, average peptide length, and amino acid composition of the protein hydrolysate impacted its interaction with polyphenols, thereby changing the antioxidant capacity of the protein–polyphenol complex [11,15,16].

Anthocyanins, a type of polyphenolic flavonoid, are abundant in several fruits, such as grape, mulberry, blueberry, cherry, and blackcurrant. Anthocyanins have been widely used as a natural food colorant; have been shown to possess several beneficial health effects, such as antioxidant, antidiabetes, anticancer, anti-inflammatory, antimicrobial, and antiobesity properties; and help in the prevention of cardiovascular diseases [17]. The most widespread anthocyanins are based on six aglycone forms, and the proportion of cyanidin is about 50% significantly higher than the other five anthocyanins (peonidin 12%, pelargonidin 12%, delphinidin 12%, malvidin 7% and petunidin 7%) [18]. Cyanidin-3-*O*-glucoside (C3G) is the most common 3-*O*-glycosidic derivative of cyanidin and the major anthocyanin among the fruits such as black elderberry, blackberry raw, black aestivalis grape, and gooseberry, and its content in fruits ranges between 0.28 and 794.13 mg/100 g [19]. Researchers [20] have used molecular modeling and multispectral techniques to investigate the binding mechanisms of C3G with myoglobin, bovine serum albumin, and hemoglobin, and obtained the binding sites, binding energy, binding forces, and binding constants of their complexes. Ren, Xiong, Li, and Li [21] indicated that glycinin had a higher binding affinity and more binding sites for C3G than β-conglycinin, and the thermal stability and surface hydrophobicity of the two proteins were reduced after interacting with C3G. He et al. [18] demonstrated that different preheating temperatures and pH influenced the C3G binding affinity and structures of milk proteins to varying degrees. Because previous research mainly focused on the binding properties of proteins and C3G, limited information is available on the interaction between protein hydrolysates and C3G. Therefore, it would be meaningful to investigate the complexation of soybean protein hydrolysates and C3G as bioactive materials in the beverage industry.

In this study, the soy protein isolate and its hydrolysates were mixed with C3G to simulate a neutral soy-based beverage with berry juice added, and the main objective of the present study was to characterize the interaction of C3G with SPI and its hydrolysates under neutral conditions and its effect on the antioxidant capacity of SPI-C3G and several SPIH-C3G (SPI/SPIHs–C3G) complexes. Multiple analytical techniques, including fluorescence quenching spectroscopy, circular dichroism (CD) spectroscopy, and Fourier-transform infrared spectroscopy (FTIR), were used to illustrate the structure changes of proteins. Moreover, we also examined the influence of the SPI/SPIHs–C3G interactions on surface characteristics, particle size, and antioxidant activity. This research offers a theoretical basis for the interactions and antioxidant activity of the hydrolysate–polyphenol complex system and helps in the design and development of functional beverages that are rich in protein hydrolysates and fruit anthocyanins.

## 2. Results and Discussion

### 2.1. Mw Distributions

The enzymatic hydrolysis of proteins is the main method for obtaining bioactive peptides [10]. Several studies reveal that the number, average peptide length, and amino acid composition of protein hydrolysates and peptides may affect their antioxidant potential and interactive properties with polyphenols [11,15]. The M_W_ distributions of SPI and SPIHs are presented in Table 1. As listed in table, the M_W_ of SPI was mostly concentrated at above 10 kDa, accounting for 87.28%, and the average Mw of the SPIHs decreased with further enzymatic hydrolysis, indicating that much smaller molecule peptides were produced. The proportions of SPIH1, SPIH2, and SPIH3 (hydrolyzing SPI for 2 min, 4 min and 15 min, respectively) with a Mw distribution above 10 kDa gradually decreased to 45.26%, 26.26%, and 12.11%, respectively, and the peak areas with an average Mw below 5 kDa progressively increased to 46.28%, 63.75%, and 81.47%, respectively. Similar conclusions have been reported by Liu, Wang, Liu, Wu, and Zhang [22] that the average Mw of hydrolysates were lower than SPI, and the proportions of comparatively low Mw (<5 kDa) distribution increased after hydrolysis by alkaline protease.

### 2.2. Intrinsic Fluorescence Spectra Analysis

#### 2.2.1. Fluorescence Quenching Mechanism Analysis

**Figure 1:**
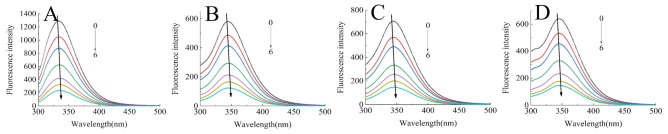
Intrinsic fluorescence spectra of 0.4 mg/mL SPI (**A**) and SPIH1-3 (**B**–**D**) in the presence of 0, 10, 20, 40, 60, 80, and 100 μM C3G (0–6) at 298 K at pH 7.0.

The intrinsic fluorescence of proteins is mainly produced by the emission of tryptophan and tyrosine residues at an excitation wavelength of 280 nm, which responds to protein denaturation, conformational transitions, and subunit association [18]. Hence, it has become a main approach to characterizing the polar microenvironment changes of proteins after interacting with polyphenols. Figure 1 presents the fluorescence spectra of SPI/SPIHs with different concentrations of C3G. It indicated that the maximum emission wavelength (λ_max_) of proteins had a red shift from 333.5 to 344.5 nm due to hydrolysis, and the fluorescence intensity of SPIHs was severely lower than SPI. Similar research also found significant red shift of λ_max_ and a reduction in fluorescence intensity in soy protein hydrolysates, which were caused by compact structure loss and the exposure of tryptophan and tyrosine residues to a more polar environment [23].

The fluorescence intensity of SPI and SPIHs apparently decreased with the increase in C3G concentration, which demonstrated that C3G quenched the intrinsic fluorescence of the proteins due to SPI/SPIHs binding. The λ_max_ of SPI/SPIHs had a slight red shift from 333.5 to 336 nm (SPI), 344.5 to 346.5 nm (SPIH1), 344 to 346 nm (SPIH2), and 345.5 to 347 nm (SPIH3) after interacting with C3G, indicating that tryptophan and tyrosine residues were in a more hydrophilic environment. These results are similar to those obtained in some related studies [16,24]. The K_SV_, K_q_ values of SPI/SPIHs with C3G are presented in Table 2 to illustrate the fluorescence quenching mechanism. The K_q_ values were much higher than the maximum diffusion collision quenching constant (2.0 × 10^10^ M^−1^ S^−1^), suggesting that the main quenching mechanism was static quenching, depending on the formation of fluorophore–quencher complexes [25].

#### 2.2.2. Binding Parameter Analysis

The K_a_ and n values of SPI/SPIHs with C3G at 298, 306, and 314 K are also listed in Table 2. The K_a_ values of the SPI/SPIHs–C3G complexes were on the order of 10^5^, and increased with the increasing temperature, suggesting that C3G had a strong binding affinity toward SPI/SPIHs and their binding reaction was endothermic [26]. Moreover, the K_a_ values of SPIHs at 298 K were lower than those of SPI, suggesting that C3G had a stronger binding affinity for SPI than its hydrolysates [27]. In addition, the K_a_ value of SPIH2 at 298 K was smaller than those of SPIH1 and SPIH3. The average peptide length and amino acid composition of the hydrolysate peptides were diverse at different times during SPI hydrolysis, which influenced its binding affinity and interaction with C3G [11]. Furthermore, the n values were approximately 1, indicating about one binding site during the interaction between SPI/SPIHs and C3G.

#### 2.2.3. Thermodynamic Parameters and Binding Force Analysis

Table 2 also includes the thermodynamic parameters between SPI/SPIHs and C3G that are related to a noncovalent type of interaction. The negative ΔG values indicated that the binding processes of SPI and SPIHs with C3G were spontaneous [18]. Moreover, ΔH > 0 and ΔS > 0 indicated that hydrophobic interactions were the predominant binding force for SPI/SPIHs–C3G complexes. The fluorescence results of Cheng, Liu, Prasanna, and Jing [28] showed that β-lactoglobulin has a strong binding affinity for C3G via hydrophobic interaction, but the molecular docking results illustrated that in addition to the multicenter hydrophobic interaction between C3G and nearby amino acid residues, C3G also established hydrogen bonds with certain residues. Therefore, we speculated that due to the involvement of the C3G active OH groups, a small part of the amino acid residues of SPI/SPIHs may also interact with C3G through hydrogen bonds, but the major binding force was still hydrophobic interaction.

### 2.3. CD Spectrum Analysis

**Figure 2:**
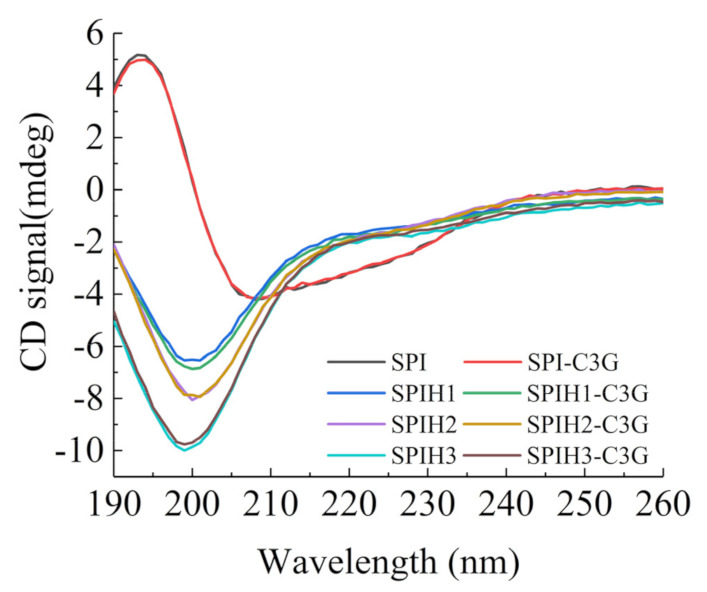
CD spectra of 100 μg/mL SPI and SPIHs with or without 1 μg/mL C3G at pH 7.0.

The CD spectra that reflect the secondary structure of SPI and SPIHs with or without C3G are presented in Figure 2. The CD spectrum of SPI revealed a broad negative peak in the vicinity of 208 nm, with an obvious positive peak near 193 nm, which suggests that the secondary structures of SPI are mainly highly ordered α and β types [29]. The considerable and progressive blue shifts of the negative peak from 208 to 201–199 nm were observed after hydrolysis, and the ellipticity of the positive signal to the negative peak was reduced, which indicated that the ordered structures (α-helixes and β-sheet) were damaged, and a random coil was formed [23].

The contents of the α-helix, β-sheet, β-turn, and random coils that were calculated using the CDNN program are summarized in Table 3. The results revealed that the interaction with C3G did not significantly change the secondary structures of SPIHs. The binding interactions with polyphenols exhibited less impact on the protein secondary structures for several noncovalent complexes of proteins and polyphenols [30]. Similar observations were also found when phenolic acids interacted with β-conglycinin [31], and when proanthocyanidin interacted with soybean seed ferritin [32].

### 2.4. FTIR Spectrum Analysis

**Figure 3:**
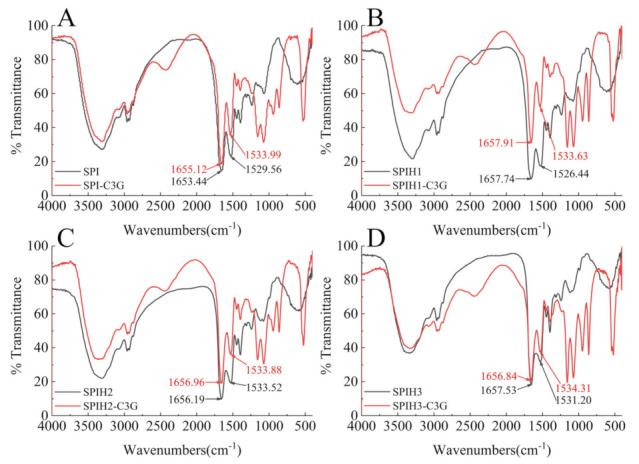
FTIR spectra of 100 μg/mL SPI (**A**) and SPIH1–3 (**B**–**D**) with or without 1 μg/mL C3G at pH 7.0.

FTIR spectroscopy was employed to further examine the secondary structure changes of proteins after binding to C3G. The spectral shifts in the amide I (mainly C=O stretch) and amide II (40% C–N stretch and 60% N–H bend) regions were mostly associated with the secondary structure changes of the proteins [25]. As an especially sensitive region, amide I was often used for protein secondary structure quantification [23]. The FTIR spectra of SPI (1) and SPIH1-3 (2–4) with or without C3G are presented in Figure 3. The amide II wavenumber shifts of SPI and SPIHs were slight, and amide I had almost no shifts (SPI: 1655.12–1653.44 cm^−1^, SPIH1: 1657.91–1657.74 cm^−1^, SPIH2: 1656.96–1656.19 cm^−1^, and SPIH3: 1656.84–1657.53 cm^−1^) after interacting with C3G. The FTIR spectra indicated that the complexation of C3G with SPI and SPIHs through hydrophobic interaction had little effect on its secondary structure, which was consistent with the CD spectra result. Chen, Li, and Tang [33] reported no observable changes in the FTIR spectra of heated and unheated SPIs after interaction with curcumin.

### 2.5. Surface Hydrophobicity (H_0_) Analysis

**Figure 4:**
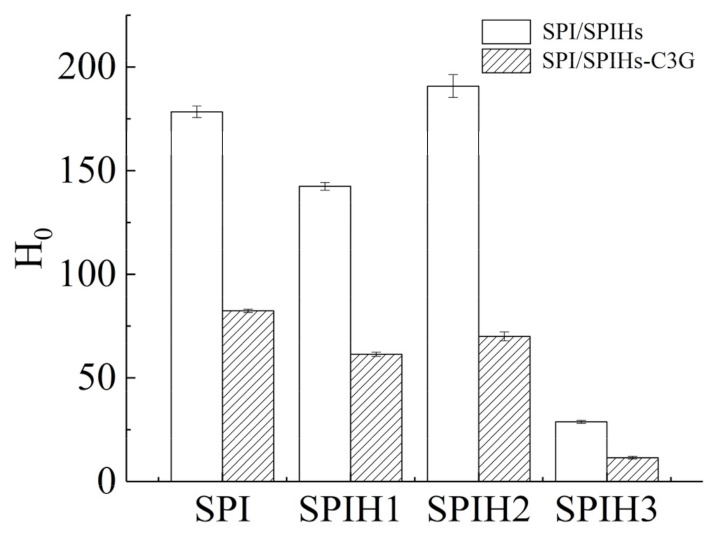
Surface hydrophobicity (H_0_) of SPI and SPIHs with or without C3G. The mass concentration ratio of SPI/SPIHs and C3G was 100:1.

H_0_ is used to characterize the number of hydrophobic sites between the surface proteins and the polar solvents, which is closely related to the conformation and functional properties of proteins [34]. The H_0_ values of SPI and SPIHs with or without C3G were quantified (Figure 4). The H_0_ of proteins revealed the various changes during SPI hydrolysis at different times, which initially decreased, then increased, and finally decreased from SPIH1 to SPIH3. The H_0_ of SPIH1 was reduced by 20.14% against SPI because several hydrophobic groups were exposed by enzymatic hydrolysis, causing protein aggregation through hydrophobic interactions [34]. As a result, the protein–protein aggregation reduced the surface area of hydrophobic groups that was exposed to the surrounding water. The 33.93% increase in H_0_ for SPIH2 in comparison with SPIH1 was caused by the steric exclusion effect produced by the aggregated proteins, which made it difficult for the further exposed hydrophobic groups to bind together. The H_0_ finally decreased by 84.90% for SPIH3 in comparison with SPIH2 because the hydrophobic areas contributing to surface hydrophobicity were broken down by enzymatic cleavage, and some hydrophilic regions that were once buried in the interior of protein were exposed to the protein surface [21,35].

The H_0_ values of SPI and SPIHs remarkably decreased after complexation with C3G, suggesting that the protein surface became more hydrophilic. C3G introduced hydrophilic groups, such as hydroxyl, which decreased surface hydrophobicity. In addition, the binding sites between ANS and the hydrophobic groups on the protein surface decreased due to the binding of hydrophobic groups to C3G through hydrophobic interactions [5,21]. Su et al. [16] also observed that as the phenolic compounds/walnut protein hydrolysates ratio (*w*/*w*) increased from 1:600 to 1:120, the fluorescence intensity of ANS almost decreased linearly, indicating a surface hydrophobicity reduction in their complexes.

### 2.6. Particle Size Distribution Analysis

**Figure 5:**
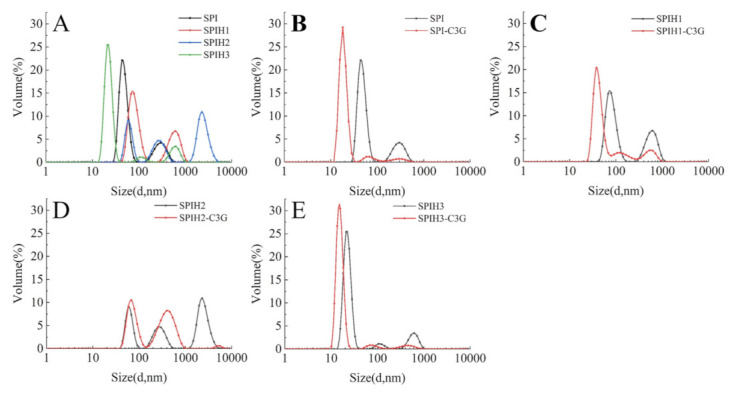
Particle size distributions of SPI and SPIHs with the absence (**A**) and presence (**B**–**E**) of C3G. The mass concentration ratio of SPI/SPIHs and C3G was 100:1.

The particle size distributions of SPI and SPIHs with or without C3G are presented in Figure 5. As displayed in Figure 5A, the particle size distributions first increased and then decreased when the hydrolysis time was increased from 0 to 15 min (SPI: approximately 43.8 nm; SPIH1: approximately 78.8 nm; SPIH2: approximately 255.0 nm; and SPIH3: approximately 21.0 nm). The initial increase in particle size can be attributed to the exposure of hydrophobic clusters or release of hydrophobic peptides, resulting in protein aggregation [33]. The particle size was reduced, because protein aggregations were broken down by further enzymatic hydrolysis, and much smaller molecule peptides were produced. These observations were in accordance with the H_0_ analysis.

As observed in Figure 5B–E, the particle size of SPI/SPIHs–C3G complexes became smaller in comparison with SPI/SPIHs alone, and the large particles of the complexes disappeared, mainly due to the strengthened interactions between SPI/SPIHs and C3G [5]. Related research also found that the addition of a high proportion of walnut phenolic compounds may form compact peptide–phenolic complexes, with a decrease in their average particle size [16]. The particle size reduction was beneficial to the stability of the beverages [3]. However, no obvious relationship was observed between the degree of hydrolysis of hydrolysates and the particle size reduction of noncovalently linked protein–C3G complexes, which may be due to the difference in the conformations, surface properties, and protein–polyphenol interactions of hydrolysates, and thus requires further confirmation.

### 2.7. Determination of Antioxidant Capacity

In this section, the in vitro antioxidant capacity of SPI/SPIHs, C3G, and SPI/SPIHs–C3G complexes was examined using two methods (ABTS and DPPH) with results presented in Table 4. The ABTS radical scavenging activity of the SPIHs gradually increased (SPIH3 > SPIH2 > SPIH1) as the enzymatic hydrolysis progressed. The increased ABTS antioxidant capacity of SPIHs was mainly due to the exposure of amino acids and bioactive peptides with antioxidant properties caused by conformational changes during hydrolysis [36]. The ABTS values of the SPI/SPIHs–C3G complexes were markedly (*p <* 0.05) larger than that of the protein alone, which could be attributed to the presence of aromatic rings and phenolic hydroxyl groups of C3G. However, the ABTS values of the SPI/SPIHs–C3G complexes were lower than the sum of their individual values, suggesting that some antioxidant groups of C3G, protein, and bioactive peptides were masked because of the protein–polyphenol interaction, which causing an antagonistic effect on the antioxidant capacity [36]. Related studies have also revealed that the interactions between proteins and polyphenols caused a masking/antagonistic effect on the antioxidant activity, preventing the antioxidants from reaching their optimum scavenging capacity in the system [8,37]. Moreover, as presented in Table 4, the relative differences between the ABTS mixed value and the sum value of SPIHs–C3G were smaller than that of SPI–C3G, suggesting that the masking effect on the ABTS scavenging capacity of SPI–C3G was stronger than that of SPIHs–C3G, which coincides with the binding affinity between SPI/SPIHs and C3G. A previous study also indicated that the binding affinity of polyphenols and proteins was positively correlated with the masking of the total antioxidant capacity of their complexes [38]. However, no obvious relationship between the masking effect of the ABTS scavenging capacity and the binding affinity of SPIHs–C3G complexes was observed. The possible reason was that the composition and structure (such as the length, amino acid composition, number of fragments, and secondary and tertiary structures) of hydrolysates influenced its interaction mechanism with the aromatic rings and phenolic hydroxyl groups of C3G, thereby affecting the results of the antioxidant capacity [15].

As can be seen in Table 4, however, there was no significant change (*p >* 0.05) in the DPPH radical scavenging activity of SPI and SPIHs, which might be because DPPH was insensitive to certain amino acids or peptides with antioxidant properties. Previous research found that the reactivity of DPPH to certain amino acids such as tryptophan and lysine was lower than that of ABTS [39]. The DPPH values of complexes were also significantly (*p <* 0.05) lower than the sum of their individual values after combining with C3G, suggesting that there was also a masking effect on the DPPH radical scavenging activity of SPI/SPIHs–C3G mixtures, similar to that of ABTS. However, unlike ABTS, there was no clear correlation between the interaction strength and the antagonistic effect on the DPPH scavenging activity of SPI/SPIHs–C3G complexes, which could be attributed to the fact that the ABTS was more sensitive toward the amino acids and peptides with antioxidant capacities than DPPH. On the other hand, it might be due to the mass ratio of SPI/SPIHs and C3G, and Su et al. [16] observed that when the phenolic compounds/walnut protein hydrolysates ratio (*w*/*w*) was over 1:12, the DPPH radical scavenging activity of the compounds showed no significant change.

In general, the interaction between SPI/SPIHs and C3G significantly (*p <* 0.05) diminished the in vitro antioxidant capacity (ABTS and DPPH) of the mixed system compared with the sum of their individual capacities.

## 3. Materials and Methods

### 3.1. Materials

Defatted soy flour with low protein denaturation was obtained from Pingdingshan Jinjing Biological Technology Co., Ltd. (Zhengzhou, Henan, China). Alcalase (200 U mg^−1^) was purchased from Shanghai Yuanye Biological Technology Co., Ltd. (Shanghai, China). C3G (purity ≥ 98%) was purchased from Nanjing Spring and Autumn Biotech Co., Ltd. (Nanjing, Jiangsu, China). 1-anilinonaphthalene-8-sulfonic acid (ANS) was obtained from Aladdin Co., Ltd. (Shanghai, China). 2,2′-azino-di-(3-ethylbenzthiazoline sulfonic acid) diammonium salt (ABTS) was obtained from Fluorochem Co., Ltd. (Hadfield, Derbyshire, UK), and 1,1-diphenyl-2-picrylhydrazyl (DPPH) was obtained from Sigma-Aldrich (St. Louis, MO, USA). Molecular weight standards including cytochrome C, aprotinin, bacitracin, Gly−Gly−Tyr−Arg, Gly−Gly−Gly, carbonic anhydrase, bovine serum albumin, alcohol dehydrogenase, β-amylase and apoferritin were all purchased from Shanghai Yuanye Biological Technology Co., Ltd. (Shanghai, China). All other reagents were of analytical grade and were purchased from Sinopharm Chemical Reagent Co., Ltd. (Shanghai, China) unless otherwise noted.

### 3.2. Preparation of SPI and Its Hydrolysates

The preparation method of SPI followed the cited previous research [40], with slight modifications. The defatted soy flour was added to distilled water at a mass ratio of 1:10 and the pH was adjusted to 8.0 with 2 M NaOH while stirring the dispersion for 2 h at room temperature (25 °C). Then, the suspension was centrifuged at 10,000× *g* for 20 min at 4 °C to obtain the supernatant. The supernatant was adjusted to pH 4.5 with 2 M HCl, kept for 30 min, and centrifuged at 3300× *g* for 10 min at 4 °C for precipitation. The precipitates were again dissolved in distilled water, and the pH was adjusted to 7.0 with 2 M NaOH while stirring the dispersion for 3 h at room temperature. The protein slurry was freeze-dried and stored at −80 °C. The protein content of freeze-dried SPI powder was 91.1%, which was measured using the Kjeldahl method (N × 6.25).

The alcalase used in this study was a kind of serine endopeptidase that consists primarily of protease from *Bacillus licheniformis*, and the optimal pH and temperature for enzymatic hydrolysis were 8.0–8.5, 50–60 °C, respectively. The SPI solutions (50 g/L) with pretreatment (10 min, 90 °C) were placed in a water bath at 60 °C, and the pH was adjusted to 8.0. Subsequently, 0.25 mg/mL alcalase enzyme was added. After hydrolysis for 2 min (SPIH1), 4 min (SPIH2), and 15 min (SPIH3), the hydrolysates were placed in a water bath at 90 °C and kept for 10 min to inactivate alcalase. Finally, the pH of the aqueous solutions was adjusted back to 7.0 and centrifuged at 9000× *g* for 20 min to obtain the supernatant. The SPIHs were freeze-dried and stored at −80 °C before use, and the water contents of SPIH1, SPIH2, and SPIH3 powders were 2.55%, 2.93%, 2.81%, respectively.

### 3.3. Determination of Molecular Weight (Mw) Distributions

The Mw distributions of SPIHs were measured using high-performance liquid chromatography (HPLC), equipped with a Waters 2487 UV detector (Waters, Milford, MA, USA) and a TSK gel G2000SWXL (300 × 7.8 mm) column placed in a 30 °C column oven. Before determination, the samples (10 mg/mL) were dissolved in a phosphate-buffered solution (PBS, 0.01 M, pH 7.0) and filtered through 0.22-μm filters (Zhejiang ALWSCI Technologies Co., Ltd., Shaoxing, China). The mobile phase consisted of a mixture of acetonitrile, ultrapure water, and trifluoroacetic acid at a volume ratio of 40:60:0.1. The flow rate was 0.5 mL/min, the sample injection volume was 10 μL, and the UV measurement wavelength was 220 nm. The MW standards were cytochrome C (12384 Da), aprotinin (6512 Da), bacitracin (1423 Da), Gly−Gly−Tyr−Arg (451 Da), and Gly−Gly−Gly (189 Da).

The determination of the Mw distribution of SPI was performed as previously described [41], with slight modifications, using HPLC equipped with a Waters 2487 UV detector (Waters, Milford, MA, USA) and a Shodex Protein KW-804 gel permeation chromatographic (GPC) (300 × 8 mm) column (Shodex Co., Tokyo, Japan) placed in a 30 °C column oven. Before determination, the sample (10 mg/mL) was dissolved in PBS (0.01 M, pH 7.0) and filtered through 0.22-μm filters (Zhejiang ALWSCI Technologies Co., Ltd., Shaoxing, China). The mobile phase consisted of PBS (0.05 M, pH 7.0) and sodium chloride (0.3 M). The flow rate was 1 mL/min, the sample injection volume was 10 μL, and the UV measurement wavelength was 280 nm. The MW standards were cytochrome C (12 kDa), carbonic anhydrase (29 kDa), bovine serum albumin (66 kDa), alcohol dehydrogenase (150 kDa), β-amylase (200 kDa), and apoferritin (443 kDa).

### 3.4. Preparation of SPI/SPIHs–C3G Complexes

The mixture of SPI/SPIHs and C3G was used as a simplified model of berry juice–soymilk beverage to investigate the protein–anthocyanin interaction and antioxidant capacity, and its preparation consisted of the following steps. SPI and SPIH stock solutions (5 mg/mL, the freeze-dried powder concentration) were prepared by dissolving lyophilized powder in PBS (0.01 M, pH 7.0) for 2 h at room temperature with moderate stirring. Subsequently, these solutions were stored overnight at 4 °C to achieve complete hydration. C3G stock solutions of two concentrations, including 1 mM for fluorescence spectroscopy and 0.25 mg/mL for other experiments, were freshly prepared just before use. The SPI/SPIHs–C3G complexes were produced by mixing the corresponding stock solutions and stirring for 2 h at room temperature. The final samples were diluted with PBS (0.01 M, pH 7.0) to meet specifications for different analytical experiments.

### 3.5. Intrinsic Fluorescence Spectra Determination

A Hitachi F-2700 fluorescence spectrophotometer (Tokyo, Japan), equipped with a xenon lamp and a 1.0-cm optical path length quartz cuvette, was used to examine the intrinsic fluorescence spectra of SPI/SPIHs with C3G at 298, 306, and 314 K. The emission spectra were recorded between 300 and 500 nm, with an excitation wavelength of 280 nm. The excitation and emission slit widths were both set at 5 nm. Moreover, the concentration of SPI/SPIHs was 0.4 mg/mL, and the concentrations of C3G were 0, 10, 20, 40, 60, 80, and 100 μM. The corresponding backgrounds of C3G and PBS were subtracted from the raw spectra of the mixtures to obtain the fluorescence parameters.

The fluorescence quenching analysis of SPI/SPIHs with C3G was conducted using the Stern–Volmer method (Equation (1)) to illustrate the mechanism of fluorescence quenching [26].

(1) $$\frac{F_{0}}{F}=1+K_{sv}\left[Q\right]=1+K_{q}\tau_{0}\left[Q\right]$$

where *F*_0_ and *F* represent the fluorescence intensities of SPI/SPIHs in the absence and presence of the quencher (C3G), *K_SV_* is the Stern–Volmer quenching constant, [*Q*] is the concentration of the quencher (C3G), *K_q_* is the bimolecular quenching constant, and *τ*_0_ is the average lifetime of the fluorophore lacking in the quencher with a value of 10^−8^ s.

If the value of *K_q_* is much larger than the maximum diffusion collision quenching constant (2.0 × 10^10^ M^−1^ S^−1^), then it suggests that static quenching (depending on the formation of fluorophore–quencher complex), rather than dynamic quenching (depending on the diffusion and collision encounters), was the main quenching mechanism [18,21]. For the static quenching mechanism, the binding constant (*K_a_*) and the number of binding sites (*n*) can be determined using a double-logarithmic equation (Equation (2)) [27].

(2) $$log\frac{F_{0}-F}{F}=logK_{a}+n\;\log\left[Q\right]$$

The type of noncovalent interaction between proteins (SPI/SPIHs) and polyphenols (C3G) was evaluated with the thermodynamic parameters calculated using the Van’t Hoff equation (Equations (3) and (4)) [27]. If ∆*H* > 0, ∆*S* < 0, then electrostatic and hydrophobic interactions predominated. If ∆*H* > 0, ∆*S* > 0, then hydrophobic interactions predominated. If ∆*H* < 0, ∆*S* < 0, then Van der Waals forces or hydrogen bonding predominated. If ∆*H* < 0, ∆*S* > 0, then electrostatic interactions predominated [26].

(3) $$ln\;K_{a}=-\frac{\Delta H}{RT}+\frac{\Delta S}{R}$$

(4) ΔG=ΔH−TΔS 

where ∆*G* is the Gibbs free energy change, ∆*H* and Δ*S* are the changes of enthalpy and entropy, *R* is the gas constant (8.314 J mol^−1^ K^−1^), and *T* is the thermodynamic temperature.

### 3.6. CD Spectroscopy Determination

The Chirascan V100 CD Spectrometer (Applied Photophysics, Leatherhead, UK) was used to measure the secondary structure changes of SPI after hydrolysis and interaction with C3G. The final concentration of SPI/SPIHs was 100 μg/mL, with or without 1 μg/mL C3G. The samples were injected into a quartz cuvette with a 1-mm path length. The scanning wavelength was from 190 to 260 nm, and the scanning speed was 60 nm/min. The spectral resolution, response time, and slit width were 0.2 nm, 0.25 s, and 1 nm, respectively. The contents of α-helix, β-sheet, β-turn, and random coil were determined using the CDNN program (Applied Photophysics, Leatherhead, UK).

### 3.7. FTIR Spectroscopy Determination

Nicolet Nexus 470 FTIR spectrometer (Thermo Electron Co., Shanghai, China) was also used to examine the secondary structure changes of SPI/SPIHs after interaction with C3G. The SPI/SPIHs solution (100 μg/mL) with or without 1 μg/mL C3G at pH 7.0 was freeze-dried for subsequent determination. The lyophilized sample was mixed with an appropriate amount of KBr and compressed into a tablet for further examination. The infrared spectra were recorded in the region from 500 to 4000 cm^−1^ at a resolution of 4 cm^−1^ with 32 scans using the Nicolet Omnic v8.0 software (Thermo Electron Co., Shanghai, China).

### 3.8. Surface Hydrophobicity (H_0_) Measurement

The protein surface hydrophobicity (H_0_) was determined using ANS. ANS, as a hydrophobic fluorescence probe, could selectively bind to exposed hydrophobic protein regions and mirror the surface hydrophobicity and conformational change in proteins [16]. The final concentrations of SPI/SPIHs were 0.5–4.0 mg/mL, with or without the corresponding concentrations of C3G ranging from 5 to 40 μg/mL (100:1). The 20 μL ANS solution (8 mM) was added to 2 mL of each diluted protein solution and kept in the dark for 3 min at room temperature (25 °C). The fluorescence intensities of the samples were measured using a fluorescence spectrophotometer (Hitachi F-2700, Tokyo, Japan) with excitation and emission wavelengths of 390 and 470 nm, respectively. The initial slope of the fluorescence intensity versus protein mass concentration plot was the value of H_0_.

### 3.9. Particle Size Distribution Measurement

Before measurement, SPI/SPIHs and SPI/SPIHs–C3G complexes (with a mass concentration ratio of 100:1) were appropriately diluted. The particle size volume distributions of the samples were determined through dynamic light scattering using a Zetasizer Nano-ZS (Malvern Instruments Ltd., Worcestershire, UK). The refractive index and viscosity of the emulsion was set to 1.33 and 0.8872, respectively, and the scattering angle was 173° backscatter.

### 3.10. Antioxidant Capacity Analysis

#### 3.10.1. ABTS Radical Scavenging Activity

The measurement of ABTS radical scavenging activity was performed as previously described [42], with slight modifications. The ABTS working solution containing 7 mM ABTS and 2.45 mM potassium persulfate was prepared away from light for 12–16 h at room temperature. Before the experiments, the ABTS working solution was diluted with PBS (0.2 M, pH 7.4) at an absorbance of 0.7 ± 0.02 at 734 nm. Then, 10 μL samples (1 mg/mL SPI/SPIHs with or without 0.04 mg/mL C3G) and 190 μL ABTS solution were transferred into a 96-well plate. After reaction in the dark for 10 min at room temperature, the absorbance was determined using a SpectraMax 190 microplate reader (Molecular Devices Corporation, San Jose, CA, USA) at 734 nm. The Equation (5) for ABTS free radical scavenging capacity was as follows:

(5) $$ABTS\;free\;radical\;scavenging\;capacity\;\left(\%\right)=\left[1-\left(\frac{A}{A_{0}}\right)\right]\times 100$$

where *A* is the absorbance of the sample, and *A*_0_ is the absorbance of the control.

#### 3.10.2. DPPH Radical Scavenging Activity

The determination of DPPH radical scavenging activity was performed as previously described [43], with slight modifications. The 100 μL sample solution (1 mg/mL SPI/SPIHs with or without 0.04 mg/mL C3G) was mixed with an equal volume of DPPH reagent (0.2 mM) in a 96-well clear flat-bottom plate and kept out of light for 30 min at room temperature, and an equal amount of buffer was used as a blank. Absorbance at 517 nm was determined using a SpectraMax 190 microplate reader (Molecular Devices Corporation, CA, USA). The Equation (6) for DPPH free radical scavenging capacity was as follows:

(6) $$DPPH\;free\;radical\;scavenging\;capacity\;\left(\%\right)=\left[1-\left(\frac{A}{A_{0}}\right)\right]\times 100$$

where *A* is the absorbance of the sample, and *A*_0_ is the absorbance of the blank.

### 3.11. Statistical Analysis

All experiments were conducted in triplicate, and the experimental results were reported as the mean ± standard deviation (SD). Statistical analysis was conducted using the general linear model procedure of the Statistix software 9.0 (Analytical Software, Tallahassee, FL, USA). The differences were considered significant when *p <* 0.05.

## 4. Conclusions

In this study, the interaction of C3G with SPI and SPIHs at pH 7.0 and its effect on the antioxidant capacity of SPI/SPIHs–C3G complexes were determined. The data revealed that C3G binds to SPI and SPIHs through hydrophobic interaction, usually with a single binding site, and the binding affinity for SPI is stronger than for its hydrolysates. Furthermore, interaction with C3G does not significantly change the secondary structures, but decreases the surface hydrophobicity and average particle size of SPI/SPIHs. Moreover, the SPI/SPIHs–C3G interactions induce an antagonistic effect on the in vitro antioxidant capacity (ABTS and DPPH) of the mixed system, and the masking effect on the ABTS scavenging capacity of SPIHs–C3G is lower than that of SPI–C3G, positively correlating with their interaction strengths. These results not only provide a theoretical basis for the interactions and antioxidant ability of the hydrolysate–polyphenol systems but could also help in the production and development of functional beverages rich in soybean hydrolysates and fruit anthocyanins at neutral conditions.

## Figures and Tables

**Table 1:** The molecular weight (MW) distributions of soy protein isolate (SPI) and its hydrolysates (SPIHs).

M_W_ Distribution (%)	SPI	SPIHs		
		SPIH1	SPIH2	SPIH3
>10 kDa	87.28 ± 0.17 ^a^	45.26 ± 0.63 ^a^	26.26 ± 0.95 ^b^	12.11 ± 0.18 ^d^
5–10 kDa	6.50 ± 0.12 ^b^	8.46 ± 0.11 ^d^	9.99 ± 0.23 ^d^	6.42 ± 0.05 ^e^
1–5 kDa	5.39 ± 0.10 ^c^	26.27 ± 0.52 ^b^	37.34 ± 1.02 ^a^	36.23 ± 1.26 ^a^
0.5–1 kDa	0.76 ± 0.01 ^d^	11.19 ± 0.29 ^c^	13.53 ± 0.49 ^c^	26.34 ± 0.68 ^b^
<0.5 kDa	0.07 ± 0.00 ^e^	8.82 ± 0.20 ^d^	12.88 ± 0.37 ^c^	18.90 ± 0.46 ^c^

Values are expressed as mean ± SD. Different superscripts (^a–e^) in the same row indicate significant differences (*p* < 0.05).

**Table 2:** The quenching constants, binding constants, number of binding sites, and thermodynamic parameters for 0.4 mg/mL SPI/SPIHs in the presence of 0, 10, 20, 40, 60, 80, and 100 μM C3G at 298, 306, and 314 K.

Sample	T (K)	K_SV_ (×10^4^ M^−1^)	K_q_ (×10^12^ M^−1^ S^−1^)	K_a_ (×10^5^ M^−1^)	n	ΔH (kJ mol^−1^)	ΔG (kJ mol^−1^)	ΔS (J mol^−1^ K^−1^)
SPI + C3G	298	4.65 ± 0.15	4.65 ± 0.15	6.01 ± 0.88 ^a^	1.29 ± 0.02	18.28	−32.97	171.98
	306	5.21 ± 0.39	5.21 ± 0.39	7.38 ± 2.04	1.30 ± 0.03		−34.37	172.08
	314	4.82 ± 0.44	4.82 ± 0.44	8.75 ± 2.49	1.33 ± 0.03		−35.72	171.98
SPIH1 + C3G	298	3.83 ± 0.09	3.83 ± 0.09	4.10 ± 0.02 ^b^	1.27 ± 0.00	66.08	−32.02	329.19
	306	4.55 ± 0.22	4.55 ± 0.22	6.28 ± 0.74	1.30 ± 0.02		−33.96	326.95
	314	4.12 ± 0.15	4.11 ± 0.15	16.02 ± 0.79	1.41 ± 0.01		−37.30	329.23
SPIH2 + C3G	298	3.85 ± 0.30	3.85 ± 0.30	2.15 ± 0.40 ^d^	1.20 ± 0.01	23.00	−30.43	179.27
	306	3.99 ± 0.15	3.99 ± 0.15	2.64 ± 0.34	1.22 ± 0.01		−31.76	178.96
	314	3.93 ± 0.04	3.93 ± 0.04	3.46 ± 0.18	1.25 ± 0.01		−33.30	179.27
SPIH3 + C3G	298	3.63 ± 0.12	3.63 ± 0.12	3.31 ± 0.63 ^c^	1.25 ± 0.02	2.53	−31.49	114.15
	306	4.02 ± 0.08	4.02 ± 0.08	3.38 ± 0.85	1.24 ± 0.03		−32.38	114.10
	314	4.27 ± 0.06	4.27 ± 0.06	3.48 ± 1.00	1.24 ± 0.03		−33.31	114.15

Values are expressed as mean ± SD. Different superscripts (^a–d^) in the same row indicate that there are significant differences in the binding constants (K_a_) of the SPI/SPIHs-C3G complexes at 298 K.

**Table 3:** CD analysis of the secondary structures of 100 μg/mL SPI/SPIHs with or without 1 μg/mL C3G at pH 7.0, determined using the CDNN program.

Samples	α-Helix (%)	β-Sheet (%)	β-Turn (%)	Random Coil (%)
SPI	16.86	34.78	19.65	28.81
SPIH1	6.73	15.54	31.49	46.34
SPIH2	6.91	20.32	29.43	43.24
SPIH3	5.46	13.01	31.48	49.95
SPI + C3G	16.73	35.00	19.62	28.65
SPIH1 + C3G	6.76	14.31	32.06	46.86
SPIH2 + C3G	6.82	20.16	29.49	43.53
SPIH3 + C3G	5.57	13.63	31.34	49.45

**Table 4:** ABTS and DPPH radical scavenging activities (%) of 1 mg/mL SPI/SPIHs with or without 0.04 mg/mL C3G.

Sample		Radical Scavenging Activity (%)	
		ABTS	DPPH
SPI		13.52 ± 0.50 ^i^	4.85 ± 0.41 ^c^
SPIH1		27.43 ± 0.73 ^h^	6.50 ± 0.48 ^c^
SPIH2		34.85 ± 2.09 ^f^	5.23 ± 0.22 ^c^
SPIH3		41.34 ± 2.19 ^e^	4.84 ± 0.21 ^c^
SPI + C3G	Mix	31.00 ± 1.13 ^g^	33.62 ± 1.74 ^b^
	Sum	41.26 ± 0.52 ^e^	38.53 ± 2.12 ^a^
SPIH1 + C3G	Mix	44.28 ± 2.03 ^e^	32.23 ± 0.53 ^b^
	Sum	55.16 ± 0.72 ^d^	40.18 ± 2.05 ^a^
SPIH2 + C3G	Mix	58.73 ± 3.97 ^c^	32.69 ± 1.87 ^b^
	Sum	62.58 ± 2.03 ^b^	38.91 ± 2.30 ^a^
SPIH3 + C3G	Mix	66.07 ± 1.85 ^a^	33.95 ± 1.21 ^b^
	Sum	69.08 ± 2.10 ^a^	38.52 ± 2.15 ^a^

Values are expressed as mean ± SD. Different superscripts (^a–i^) in the same row indicate significant differences (*p* < 0.05). Mix denotes the ABTS/DPPH values of the mixture solutions of SPI/SPIHs and C3G. Sum denotes the sum of the ABTS/DPPH values of the individual SPI/SPIHs solutions and C3G solution.

## Data Availability

The data presented in this study are available in the article.

## Sample availability

Samples of the compounds are available from the authors.
